# Biomarkers in Colorectal Cancer: Current Research and Future Prospects

**DOI:** 10.3390/ijms21155311

**Published:** 2020-07-27

**Authors:** Olorunseun O. Ogunwobi, Fahad Mahmood, Akinfemi Akingboye

**Affiliations:** 1 Department of Biological Sciences, Hunter College of The City University of New York, New York, NY 10065, USA; 2 Joan and Sanford I. Weill Department of Medicine, Weill Cornell Medicine, Cornell University, New York, NY 10021, USA; 3 The Dudley Group Hospitals, Russells Hall Hospital, The Dudley Group NHS Foundation Trust, Dudley, West Midlands DY1 2HQ, UK; fahad.mahmood@nhs.net

**Keywords:** *PVT1*, colorectal cancer, screening, diagnosis, prognostication, biomarker

## Abstract

Colorectal cancer (CRC) is a leading cause of death worldwide, despite progress made in detection and management through surgery, chemotherapy, radiotherapy, and immunotherapy. Novel therapeutic agents have improved survival in both the adjuvant and advanced disease settings, albeit with an increased risk of toxicity and cost. However, metastatic disease continues to have a poor long-term prognosis and significant challenges remain due to late stage diagnosis and treatment failure. Biomarkers are a key tool in early detection, prognostication, survival, and predicting treatment response. The past three decades have seen advances in genomics and molecular pathology of cancer biomarkers, allowing for greater individualization of therapy with a positive impact on survival outcomes. Clinically useful predictive biomarkers aid clinical decision making, such as the presence of *KRAS* gene mutations predicting benefit from epidermal growth factor receptor (EGFR) inhibiting antibodies. However, few biomarkers have been translated into clinical practice highlighting the need for further investigation. We review a range of protein, DNA and RNA-based biomarkers under investigation for diagnostic, predictive, and prognostic properties for CRC. In particular, long non-coding RNAs (lncRNA), have been investigated as biomarkers in a range of cancers including colorectal cancer. Specifically, we evaluate the potential role of lncRNA plasmacytoma variant translocation 1 (*PVT1*), an oncogene, as a diagnostic, prognostic, and therapeutic biomarker in colorectal cancer.

## 1. Introduction: Epidemiology, Burden of Disease and Challenges in Treatment & Chemoresistance

### 1.1. Epidemiology

Colorectal cancer (CRC) is the fourth most common cancer overall worldwide contributing to 9.7% of global cancer burden [1,2,3,4,5]. It affects 746,000 men (10% of all cancer cases) and 614,000 women (9.2% of all cancer cases) with most cases (55%) occurring in developed countries [3,6]. Furthermore, in the UK, 42,300 new colorectal cancer cases are diagnosed each year making it the fourth most common cancer overall, and third most common in males and females [5]. In addition, the incidence of colorectal cancer increased between 1991 and 2016 and is attributed to lifestyle, environmental changes, and aging populations [7,8]. Although bowel cancer incidence has fallen in the UK in the past decade by 4%, the lifestyle risk factors remain. Moreover, the burden of CRC is expected to increase with 2.2 million new cases and 1.1 million deaths expected globally by 2030 [9]. In addition, significant challenges remain in managing disease burden. In England, five-year overall survival for CRC is 58.4% which is lower than the US reported 60–65% [10,11]. Moreover, US reported survival was static between 1996 and 2014 [10,11]. Further challenges remain due to the ageing demographic and advanced presentation of disease [12]. Older patients, above age 75 years, make up 44% of new colorectal cancer diagnoses and an estimated 20–25% of CRC is diagnosed at the metastatic stage with an additional 25% of patients developing metastasis during their illness [5,13]. As a result, CRC accounts for 8.5% of cancer related deaths worldwide with 16,300 deaths per year in the UK making it the second most frequent cause of cancer related deaths at 10%. Although survival is stage dependent with 92% survival for stage I, compared to 10% in stage IV, there has been an improvement in survival for the 60–69 year age group attributed to screening [5]. Thus, CRC remains a prevalent challenge in cancer management emphasizing the need for early diagnosis. 

### 1.2. Screening Programs

Declining mortality due to improvements has been shown with early detection through screening and effective treatment [14,15,16,17]. The UK CRC screening program relying on fecal detected occult blood (FOBT) and colonoscopy has led to 16% decline in overall mortality rate without affecting incidence [18]. However, FOBT has reduced sensitivity for advanced adenomas and CRC which may improve with newly implemented immunochemical testing (FIT) [19,20]. This screening test is offered in the UK every 2 years between 60–74 years with a one-off test aged 55 years [5]. These tests are precursors for more invasive colonoscopy to identify pre-malignant or malignant lesions. Furthermore, studies randomized trials have shown a reduction in CRC incidence up to 23% and CRC-related mortality by 31% using flexible sigmoidoscopy as a primary screening tool [21]. However, this remains an invasive and resource intensive technique. There is no universally agreed screening protocol for early disease stages, and significant variation remains. In addition, up to 70% of cancers presenting with symptoms are at an advanced stage [22]. This emphasizes the value of screening programs with early detection of pre-malignant or early stage (I-II) CRC leading to improved CRC survival, quality of life and disease-free outcomes. Moreover, screening for biomarkers at all stages, including diagnostic, prognostic, and predictive, may provide opportunities for targeted intervention to improve outcomes whilst reducing the risk of treatment toxicity [10,22]. 

### 1.3. Current Treatment Effectiveness

Treatment of CRC depends upon stage of disease according to the TNM classification, patient health, and curative versus palliative intent [12,23,24,25]. This comprises surgery, chemotherapy, and immunotherapy. Factors including stage, circumferential resection margin, lymphovascular invasion, perineural invasion, and genotyping are used to determine need for and type of adjuvant treatment [26,27]. Fluorouracil (5-FU), a fluoropyrimidine is used as part of the FOLFOX (Folinic acid + Oxaliplatin) or FOLFIRI (Folinic acid + Irinotecan) regimens leading to improved overall disease-free and progression-free survival in both advanced and metastatic disease [24,27,28,29]. However, 5-FU is associated with toxicity and reduced clinical response in patients with microsatellite instability (MSI) status as well as dihydropyrimidine dehydrogenase (DPYD) deficiency [30,31,32,33,34]. In addition, 5-FU leads to a modest 2–4% improvement in five-year disease free survival in stage II CRC [35]. However, previous studies have shown between 20–25% recurrence in treated Stage II lymph node negative colon cancer within five years [36,37]. In addition, the anti-EGFR cetuximab and anti-vascular endothelial growth factor receptor (VEGFR) bevacizumab response rate is higher in Kirsten rat sarcoma viral oncogene (*KRAS)* wildtype compared to *KRAS* mutants leading to its application in clinical practice [38,39]. Moreover, in metastatic CRC, the anti-programmed cell death receptor-1 agents Nivolumab and Ipilimumab have shown benefit in MSI and mismatch repair deficient genotypes thereby gaining approval in patients progressing on first line chemotherapy [40]. Further experimental treatments such as Regofarenib, an anti-angiogenic compound, shows poor overall survival in *KRAS* mutants but improved progression free survival in association with phosphorylated proline-rich protein kinase B (AKT) in metastatic CRC [41,42]. Thus, although the revised TNM application may lead to a reduction in over or undertreatment of CRC, the risks versus benefits of treatment selection need to be informed by molecular characteristics of individual tumors to develop personalized treatment, overcome poor efficacy and chemoresistance. 

## 2. Why Do We Need a Biomarker: The Role for Biomarkers in Early Detection of Colorectal Cancer

Biomarkers are molecular patterns that can be used as a tool for early cancer detection and individualized CRC treatment [30,43,44]. They can be divided into diagnostic, prognostic, or predictive categories. Thus, biomarkers provide utility at different stages of the disease to determine disease progression, recurrence, as well as providing a personalized indicator for therapeutic effectiveness. 

Firstly, early diagnosis in asymptomatic patients remains a key target to achieve favorable survival outcomes through identification of early CRC as well as pre-malignant lesions including high risk polyps. The sensitivity for detecting CRC using current FIT testing (100ng/mL) is 73.8% versus 92.3% for a stool-based DNA assay screening *KRAS*, aberrant *NDRG4* and *BMP3* methylation [19]. Furthermore, FIT testing sensitivity for advanced precancerous lesions is 23.8% versus 42.4% with stool DNA testing [19]. Moreover, the rate of detection of polyps with high-grade dysplasia is 46.2% with FIT testing verses 69.2% with stool DNA testing, whereas the detection rate of serrated sessile polyps measuring >1 cm is only 5.1% (FIT) versus 42.4% with stool DNA sampling [19]. These findings highlight the limits of current diagnostic screening and difficulty in establishing appropriate surrogate markers for early disease detection. Current non-invasive screening stools are not sensitive to detect pre-cancerous lesions and may miss significant early CRC. A low threshold must therefore be maintained for more invasive colonoscopy in these patients and further tools are required to support identifying early CRC. 

Secondly, prognostic biomarkers can be used to predict disease progression including early recurrence and mortality [10,44]. KRAS is part of the *RAS* proto-oncogene family of GTPases which acts to turn off cell proliferation [45]. Mutations in *KRAS* are associated with increased risk recurrent metastatic CRC following curative resection as well as worse overall survival following hepatic metastasectomy in metastatic CRC [46,47]. Furthermore, the *BRAF* proto-oncogene works via the RAS-RAF-MEK-ERK pathway regulating cell transcription [48]. The *BRAF* V600E mutation is associated with reduced survival, including progression-free and up to 50% worse overall survival compared to *BRAF* wildtype [49,50,51,52]. In the emerging field of radiogenomics, a combination of radiological and genetic features may give greater prognostic sensitivity than either of these modalities in isolation [53,54]. Finally, the carcinoembryonic antigen (CEA), a high molecular weight glycoprotein is used as a biomarker to predict early recurrence in post-operative patients despite low sensitivity and specificity [55,56]. Thus, using prognostic markers may alter thresholds for further investigation of recurrent disease and provide opportunities for early intervention. Moreover, they may alter thresholds at which patients are offered more aggressive treatment. 

Additionally, predictive biomarkers are used to individually tailor treatments according to molecular subtype. *KRAS* mutations are associated poor response to anti-EGFR receptor therapy including cetuximab and panitummumab [57,58]. There was a 16% increase in overall response rate in *KRAS* wildtype patients with FOLFIRI and cetuximab compared to 4% decrease in *KRAS* mutants. Since *KRAS* mutantions are present in up to 40% of patients, a significant portion of patients can be spared expensive anti-EGFR treatment. Furthermore, irinotecan, a topoisomerase inhibitor used as part of FOLFIRI regimen, is metabolized by diphosphate-glucuronosyltransferase 1A (UGT1A). Homozygosity for UGT1A1*28 allele is associated with dose dependent increase in toxicity compared to UGT1A1*1 genotype [59]. Moreover, dihydropyrimidine dehydrogenase (DPD) is responsible for metabolizing more than 80% of 5-FU [56]. DYPD*2A and DPYD*13 variants lead to increased toxicity with evidence that reducing 5-FU dose by 25–50% can lead to a reduction in toxicity [60]. These interventions may thus lead to improved treatment response and reduced toxicity arising from ineffectual interventions. They can also help in making dose adjustments to gain maximum benefit from a selected regimen. The need to develop further biomarkers is amplified by the fact that only *KRAS*, *NRAS*, *BRAF* and MSI status is recommended by national guidelines in evaluating treatment response and predicting outcomes in CRC [61]. However, several potential categories of biomarkers remain under investigation. 

## 3. Recent Advances in the Molecular Subtyping of Colorectal Cancer and Its Implication on Personalised Gene Therapy

Molecular subtyping of colorectal cancer has led to several categories of potential biomarkers through somatic mutations, germline pharmacogenomics, cancer stem cells, microRNA, and long non-coding RNA (lncRNA) [10,56,62,63].

### 3.1. Somatic Mutations

Somatic mutation relies on identification of genes in known signalling pathways associated with CRC progression. Combined inhibition of MEK, a downstream target of the RAS/MAPK pathway with binimetinib, along with encorafenib (BRAF inhibition) and cetuximab (EGFR inhibition) to *BRAF* V600E mutants who had progressed on previous chemotherapy showed overall response rate of 48% and progression free survival of eight months vs. two months [64]. Moreover, *MEK* inhibitor resistant CRC cell lines show increased expression of the WNT signalling pathway with evidence of anti-tumor response with cyclosporin A and selumetinib [65,66]. However, there no evidence that *MEK* status has prognostic value, or that it leads to an improved response in *KRAS* mutant phenotypes not sensitive to anti-EGFR therapy although further evidence is required for predictive utility. Furthermore, amplified human epidermal growth factor receptor 2 (*HER-2*) gene is present in 3% of CRC patients [67]. The use of transtuzumab and lapatinib on *KRAS* wildtype and *HER-2* amplified genotype that progressed on prior therapy led to a 70% clinical benefit and overall response rate of 30.3% in the HERACLES trial [68]. In addition, Phosphatidylinositol-4,5-bisphosphate 3-kinase catalytic subunit alpha (*PIK3CA*) part of the PI3K/AKT/mTOR pathway has mutations in exon 9 or exon 20 in 10–15% cases [69]. In the VICTOR randomised trial, recurrence free survival was lower in stage II and III CRC after resection if taking 100mg aspirin per day [69]. Moreover, mutations in *PTEN*, a tumor suppressor gene and also part of the PI3K/AKT/mTOR pathway, may lead to poor survival and increased risk of metastatic disease [70,71]. Shen et al. showed an improved response to cetuximab with wildtype *PTEN* although the prognostic value of *PTEN* has not been corroborated by further studies [70,72]. In addition, several prognostic markers have been evaluated. CpG island methylator phenotype (CIMP) displays hypermethylation in tumor suppressor or tumor-related genes which can be categorized as CIMP-High or CIMP-Low [73,74]. CIMP positive tumors display worse overall and disease-free survival with differential response to standard chemotherapeutic agents including 5-FU and irinotecan [75]. However, CIMP tumors often have concomitant mutations in *BRAF* or MSI making it difficult to interpret the contribution of CIMP as an independent prognostic marker [76]. Detection of DNA methylation aberrations in cell-free DNA has been proposed to detect early changes in the pathogenesis of CRC [77]. For example, hypermethylation of septin-9 (*SEPT9*) has a sensitivity of 51–90% and specificity of 73–96% in the serum of CRC patients [77]. However, sensitivity of *SEPT9* for advanced adenomas is only 9.6% limiting utility as a predictive biomarker. Further hypermethylation aberrations have been investigated in cyclin dependent kinase 2A, *LINE-1*, *MLH1,* and *APC* genes complementing mutational analysis although none have been validated. Furthermore, DNA aneuploidy, a surrogate marker of chromosomal instability, is associated with poor overall survival in stage II and III CRC as well as risk of early relapse in stage II CRC [78,79]. Moreover, loss of chromosome 18q, associated with *DCC*, *SMAD4*, *SMAD2,* and *CABLES1* tumor suppressor gene inactivation leads to poor overall CRC survival [80]. A meta-analysis highlighted survival in stage II CRC was 54% vs. 83% in the presence of 18q deletions. In addition, over-expression of the pro-apoptotic *BCL-2* gene is associated with an improved overall and disease-free survival in CRC, highlighting its potential as a prognostic marker [81]. Finally, there is limited evidence that quantification of tumor infiltrating lymphocytes (TILs) and mutations in the *POLE* gene encoding the DNA Polymerase epsilon subunit, may have prognostic value in overall or disease-free survival [56]. 

### 3.2. Germline Pharmacogenes

A second category of biomarkers are alterations in the germline pharmacogenes [56]. The utility of germline mutations has been shown in predicting CRC risk. In particular, mutations in the adenomatous polyposis coli (*APC*) gene lead to familial adenomatous polyposis syndrome with a 100% risk of cancer progression without intervention. Moreover, mutations in *MLH1* and *MSH2* mismatch repair genes are associated with Lynch syndrome [82]. In addition, polymorphism 2R/3R of thymidylate synthase gene (*TYMS*), a target of 5-FU, leads to increased toxicity as per the QUASAR2 study [56]. Furthermore, methylenetetrahydrofolate reductase (MTHFR) has a role in re-methylation of homocysteine to methionine. Reduced enzyme activity causes accumulation of 5,10-MTHF which forms a complex with FdUMP (metabolite of 5-FU) to stop DNA synthesis, thereby theoretically increasing 5-FU mediated toxicity [56,83]. MTHFR 1298A > C and 677C > T diplotypes were associated with increased risks of 5-FU chemoradiation mediated toxicity in one trial for rectal cancer [84]. Furthermore, *VEGFA* polymorphism rs833061 with genotypes TC and TT have shown improved progression free survival in CRC treated with FOLFIRI and bevacizumab [85]. Moreover, *VEGFA* polymorphisms may have prognostic implications due to its potential role in lymphatic spread. Finally, the TOSCA trial examined the impact of selected polymorphisms of nucleotide excision repair (NER) enzymes but did not find an association with clinical outcome or toxicity in CRC [86]. Thus, identification of relevant mutations in target genes can lead to a personalized therapeutic regimen that may improve overall or disease-free survival. However, no targets have yet gained appropriate prospective validation or widespread clinical acceptance. 

### 3.3. Cancer Stem Cells

Other categories of biomarker remain at early experimental stages. Overexpression of several stem cell markers are associated with CRC. Oncogene B cell specific moloney leukemia virus integration site-1 (*BMI-1*) has a role in regulation of stem cell renewal [87]. Overexpression of *BMI-1* is associated with a poor prognosis in CRC and may be associated with an increased response to chemotherapeutic agent paclitaxel [88]. Furthermore, *LGR5* and *EPHB2* have shown increased expression in CRC mucosa and are associated with greater CRC relapse risk [89]. Although stem cell markers may predict tumor growth, metastasis and recurrence, there is no validated candidate marker. In addition, circulating DNA and cell free DNA provide an easily accessible biomarker source for monitoring disease progression and recurrence. Circulating DNA may be indicative of residual disease in CRC and other malignancies including breast where it may serve as potential biomarker for *BRCA1* or *BRCA2* mutation carriers as well as a surrogate marker for progression-free survival [90,91,92,93,94]. This in turn may be associated with an increased risk of recurrence and has the potential to inform adjuvant treatment decisions. Consistent with this, detecting circulating free DNA released from cell death is associated with reduced recurrence-free and overall survival in metastatic CRC [95]. Moreover, suppression of circulating DNA could be used as a surrogate marker for treatment response although this remains to be validated. In addition, circulating free DNA can be used as a surrogate marker for *BRAF* and *KRAS* mutations in place of tissue sampling. According to the ColoBEAM study there was a concordance of 89.3% between blood and tissue for *RAS/BRAF* status [96]. Thus, several potential prognostic and predictive targets have been identified, however there is little evidence from prospective trial data or validation studies to warrant clinical application as biomarkers. Moreover, biomarker sampling can be based on a tissue sampling as well as more easily accessible sources, including blood, urine, and faeces. Potential techniques relying on detection of DNA, RNA, and protein biomarkers in serum, faeces, and other non-invasive sampling methods provide a route for both rapid and accurate screening. Moreover, single biomarker studies have been limited by sensitivity and specificity which may be improved by combining several related markers to better prognostic and predictive value.

## 4. Recent Advances in the Understanding of the Biogenesis of MicroRNAs/Long Non-Coding RNAs as Potential Biomarkers in CRC 

### 4.1. MicroRNAs and Colorectal Adenoma

MicroRNAs (miRNAs) are 18–25bp ribonucleotides that bind to the 3′ end of mRNA inhibiting translation [97]. They regulate gene expression through the regulation of translation affecting cell growth, differentiation, and apoptosis [97]. miRNAs are implicated in regulating pathogenesis in cancer by incorporating oncogenic and tumor suppressor functions through various transcripts. These extend to regulation of cell migration, invasion, and metastasis as well as immune system interactions and angiogenesis affecting CRC disease progression through epithelial to mesenchymal transition activation [98,99,100]. Furthermore, expression of miRNAs can be detected in plasma, stool, urine, saliva, and tissue samples which is enhanced by their relative stability against endogenous RNAses [101]. These nucleotides have therefore been investigated for diagnostic, prognostic, or predictive qualities in CRC. 

### 4.2. MicroRNAs and Colorectal Cancer

Several studies have examined the expression pattern of miRNAs at early and advanced stages of CRC. Elevated plasma miR-92a and miR-29a is associated with advanced adenomas with 62.2% sensitivity and 84.7% specificity for miR-29a and 64.9% sensitivity and 81.4% specificity for miR-92a [102]. Moreover, combined sensitivity and specificity was greater than individual values. miR-760 and miR-601 have been shown to be reduced in advanced adenomas whereas a panel of miR-532–3p, miR-195, miR-331, miR-17, miR-142-3p, miR-15b, miR-532, and miR-652 can distinguish advanced adenomas with 88% sensitivity and 64% specificity [103]. Other panels showing elevated miR-21, miR-29a and miR-125b expression have been correlated with early CRC as well as distinguishing advanced from non-advanced CRC [104]. Moreover, these miRNAs were elevated in high-grade intra-epithelial neoplasms as well as tubular adenomas. Furthermore miR-141 elevation has been associated with advanced stage IV CRC with reported sensitivity of 66.7% and specificity of 84% in discriminating from stage I-II CRC [105]. Elevated miR-29a has been associated with early detection of liver metastasis [106]. In addition, elevated miR-221 has shown prognostic value and is associated with poor overall CRC survival and correlates with p53 expression [107]. Elevated miR-203 has been correlated with higher TNM stage, lymph node, peritoneal, and distant metastasis with resulting poor overall survival [108]. Several miRNAs have thus been associated through increased or decreased expression with poor prognosis either as individual markers or as panels. Finally, miR-19a has been associated with poor response and chemoresistance to FOLFOX regimen [109]. Conversely miR-204-5p elevation is associated with 5-FU sensitivity due to downregulation of *RABB22A*, part of the *RAS* oncogene family [110]. Moreover, five miRNAs: miR-20a, miR-130, miR-145, miR-216 and miR-372 are significantly reduced, showing 92% sensitivity and 88% specificity, in selecting for oxaliplatin resistance [111]. Elevated miR-126 was predictive of poor response to bevacizumab with decreased levels post-treatment associated with improved survival [112]. Interestingly, an increase in miR-155-5p of >30% after one month of bevacizumab treatment in advanced CRC was predictive of improved progression free and overall survival [113]. This opens the possibility for monitoring treatment response as well as predicting treatment efficacy. Thus, several further cell-free and exosomal miRNAs derived from stool, plasma and tissue have shown altered expression either individually or as part of larger panels. These miRNAs may show greater sensitivity and specificity for diagnostic, prognostic or predictive purposes if combined with other microRNAs or other forms of biomarker. However, much of the data from these studies are preliminary with limited prospective validation studies in sufficiently large cohorts. Moreover, owing to the heterogeneity and non-standardized application of study protocols, there is some inconsistency in the reported associations of some miRNAs which will need to be evaluated. 

### 4.3. Long Non-Coding RNAs

Long non-coding RNAs (lncRNA) are non-translated RNAs transcripts of at least 200 nucleotides in length accounting for 68% of the human transcriptome [114]. They are involved in a range of cellular processes including regulation of transcription, post-transcriptional control of mRNA, protein stability, subcellular structural organization, and epigenetic regulation [62,63,115]. These processes are critical in cell proliferation, migration, and survival [63]. Furthermore, they show differential distribution between cell and tissue types. Several lncRNAs have been investigated for roles in tumorigenesis or tumor suppression. 

Tumor suppressor functions of lncRNAs can be mediated by interactions with tumor suppressor gene *p53* [62]. The lncRNA activator of enhancer domains (*LED*) is up-regulated by *p53* and downregulated expression is associated with CRC, breast cancer and androgen-sensitive prostate cancer [116,117]. Furthermore, depletion of the co-activator of p21 lncRNA (lincRNA-p21) accelerates cell proliferation whereas overactivation impairs cell proliferation in diffuse b-cell lymphoma with higher levels correlating with progression-free and disease-free survival [118]. Moreover, in CRC, higher levels of lincRNA-p21 enhance sensitivity to radiotherapy, promoting apoptosis whereas lower levels lead to increased disease progression [119]. DINO, a lncRNA which participates in the p53 dependent DNA-damage response, shows low expression in CRC cell lines [120]. Further lncRNAs implicated in CRC include *NEAT1*, a p53 target gene, which in response to stress forms paraspeckle complexes that are associated with poor prognosis [117]. In addition, several lncRNAs have been implicated in driving CRC tumor growth including increased *MALAT1* expression, which is implicated in poor prognosis and metastasis [121,122]. Furthermore, HOTAIR lncRNA expression is involved in CRC tumor progression and decay may be associated with enhanced radiosensitivity [123]. Thus, lncRNA expression is at the early stages in use as a biomarker for risk of disease progression, survival, and as a therapeutic target. 

## 5. The Role of PVT1 in the Diagnosis, Treatment and Prognosis of Colorectal Cancer

Plasmacytoma variant translocation 1 (*PVT1*) is a lncRNA located on human chromosome 8q24.21 adjacent to the oncogene *C-MYC* and undergoing p53 dependent transcription [124,125]. It consists of 1957 base pairs encoding between nine and 12 exons that are variably spliced along with introns giving rise to six miRNAs: miR-1204, miR-1205, miR-1206, miR-1207-3p, and miR-1207-5p [124,126]. Moreover, at least 14 alternately spliced transcripts have been identified at tissue-detectable levels with 11 transcripts present in normal gastrointestinal mucosa as well as adenocarcinoma [115]. The *PVT1* gene is differentially expressed among populations [127]. Furthermore, quantification of *PVT1* expression pattern reveals variations between tissue types with maximal expression in ovaries, lymph nodes and bone marrow and moderate levels of expression in the colon [128]. Of note, the PVT1-217 transcript is the most abundant in the gastrointestinal tract mucosa [115]. Furthermore, *PVT1* expression is elevated in multiple cancer types including lung [129], prostate [130], cervical [131], and colon [132]. Possible functional roles for *PVT1* are mediated by miRNAs, and competing endogenous RNA (ceRNA), involving regulation of gene activity through *C-MYC* activation [115,125,133]. There is evidence of *PVT1* acting as a tumour-suppressor DNA boundary element through competition with the C-*MYC* promoter for shared enhancers within the gene locus [133]. Moreover, *PVT1* activity may affect cell growth, replication and proliferation which may drive both carcinogenesis and chemoresistance [134]. 

Several studies have shown a potential oncogenic role for *PVT1* [135] with implications for tumor initiation, progression, spread and survival. Takahashi et al. examined cell lines from 164 CRC patients, showing an increase in *PVT1* expression in tumor cells which correlated with poor overall survival. Moreover, knockdown of *PVT1* with siRNA promoted apoptosis and reduced the invasive capability of cells [136]. High expression of specific splice variants like *PVT1* -214 is associated with poor overall survival and acquisition of stem-cell like properties including invasion and cell migration [137]. Furthermore, downstream targets of *PVT1* such as miR-26b could provide both a mechanism as well as more specific biomarker readouts of *PVT1* activity in CRC [128]. Poor overall survival with elevated *PVT1* expression as well as increased cell proliferation, invasion and metastasis has been shown in further studies [138]. In addition, high relative levels of *PVT1* in extracellular vesicles from CRC cell lines SW480 and SW620 with higher levels in the more aggressive SW620 line [139]. This was associated with co-amplification of *C-MYC* and C-MYC dependent genes *FUBP1*, *EZH2*, and *NPM1*. Moreover, this effect was reversed with inhibitory siRNA resulting in an increase in apoptosis and reduction in cell proliferation. Finally, quantification of *PVT1* expression from tumors and adjacent normal tissue in 210 CRC patients showed a 51.4% increase correlating with tumor differentiation, invasion, higher stage, and lymph node spread [140]. High *PVT1* expression in these patients was associated with reduced overall and disease-free survival. Interestingly, not all CRC cell lines show invasive behavior attributable to *PVT1*. The HCT116 CRC cell line did not show greater invasiveness compared to control lines [141]. Overall, the correlation of high *PVT1* expression and reduced overall survival in CRC as well as other types of cancer has been encapsulated in a meta-analysis of 39 studies [142]. Another promising area is the identification of *PVT1* polymorphisms which predict outcomes in CRC. The rs1252200336 polymorphism showed a 2.71 times higher risk of CRC in the ID vs. II genotypes with lower survival in the Han Chinese population [143]. Thus, *PVT1* has the potential to be a prognostic biomarker in CRC that correlates with disease severity and aggressive phenotypes. Much of the work however has been done in cell-based assays which will need to be replicated in clinical settings. Table 1 summarises the current literature explaining the oncogenic role of *PVT1* through its actions on miRNAs in promoting CRC. 

*PVT1* expression can be used as a readout of therapeutic drug response as well as drug resistance. In a comparison of cisplatin sensitive versus resistant CRC patients, overexpression of *PVT1* was associated with cisplatin resistance [144,145]. This was mediated by upregulation of multi-drug resistance protein 1 (MRP1) and inhibition of the intrinsic apoptotic pathway with decrease in *BCL-2* expression. These changes could be reversed by siRNA targeting *PVT1*. Furthermore, the HCT116 CRC cell line resistant to 5-FU displays high levels of *PVT1* expression and upregulation of MRP1 [141,146]. siRNA against *PVT1* led to reduced cell survival and increased apoptosis as well as reduced MRP1 expression [146]. Similar findings have been demonstrated within in vitro models showing 5-FU resistance with high PVT1 expression in gastric cancer [147,148] and glioma [135]. Therefore, *PVT1* expression can be used as a biomarker to rationalize treatment selection in CRC patients by predicting drug resistance. Moreover, *PVT1* may itself be a target for therapeutic intervention [144]. 

Finally, *PVT1* has the potential to be a diagnostic biomarker although few studies have investigated this potential. Gharib et al. investigated *PVT1* expression as a biomarker of lymph node metastasis but noted a higher AUC when combined as part of panel of biomarkers including *PVT1*, *HOTTIP* and *UCA1* expression [149]. Currently, no studies have investigated the potential for *PVT1* expression as a biomarker for earlier stages of CRC. This in part is limited by lack of data on temporal variation with disease progress particularly within in vivo models. 

## 6. Discussion and Future Perspectives

Colorectal cancer remains a major cause of mortality worldwide. Early diagnosis is key to improve overall survival, reduce disease-free progression and reduce risk of recurrence. Biomarkers play a key role in early disease identification, can help predict disease progress and response to treatment. The management of CRC is facilitated by biomarkers used at various stages of the disease, but each have their limitations. Moreover, development and validation of biomarkers through identification of candidates must progress through understanding molecular interactions facilitating pathogenesis. Currently, there are no universal markers identifying patients at risk of invasion, lymph node metastasis, or treatment resistance to current therapeutic regimens. 

*PVT1* an oncogenic lncRNA has shown association with increased risk of tumor invasion, advanced stage and poor overall survival in multiple different cancer types including CRC. It may also be involved in chemoresistance to medications commonly used to treat CRC. *PVT1*s relative stability against endogenous RNases may facilitate utilization as a biomarker. Despite this, *PVT1* need to overcome challenges in order to establish itself as a diagnostic, prognostic, or therapeutic biomarker. Variation of *PVT1* during the course of CRC is yet to be determined along with amenability of the lncRNA to liquid biopsies to yield clinically relevant variations in expression. Liquid biopsies of surrogate biomarkers will facilitate patient comfort by circumventing the need for repeat biopsies along with enabling monitoring treatment response [44]. In particular, these can assess minimal residual disease, monitor drug resistance over time, risk of relapse or metastasis and assist in more accurate staging to avoid under or over treatment protocols. For example, a five-gene methylation panel from cell-free circulating DNA in liquid biopsies could be used to predict overall and progression free survival in 182 patients [157]. Analysis of mutations in cell-free circulating DNA has been used as a surrogate to evaluate treatment response in CRC [158]. In addition, future studies need to look at panels or combinations of biomarkers including RNA, DNA, and protein assays, combined with current biomarkers as necessary to enhance sensitivity and specificity. A further limitation is understanding the relevance of PVT1 in CRC pathogenesis. Most studies have examined *PVT1* expression without focus on particular splice variants. Most *PVT1* splice variants are generated from intronic sequences, which will require adaptation of inhibitory RNA technology for specific intra-nuclear targeting [115]. Given the potential differences in target sites and mechanisms of action, this may impact on prognosis as well as drug resistance. Understanding downstream targets of *PVT1* action may facilitate the development of validation assays including WNT, TGF-beta and p53 pathways. Finally, the potential for *PVT1* to facilitate individualized biomarker-informed therapy is promising. This will require further in vivo studies on the spectrum of chemotherapeutic agents to predict response which requires the development of animal models. 

## 7. Conclusions

The approach to patients with colorectal cancer has evolved due to an improved understanding of carcinogenesis and advances in the field of genetics. The determination of *KRAS*, *BRAF*, and MSI status has become an indispensable step in therapeutic planning, especially in patients with metastatic disease. However, even with these advances, there is a lack of biomarkers that can guide the early diagnosis, targeted treatment, prognosis, and surveillance of patients with colorectal cancer. For this reason, we believe that understanding *PVT1* could play the role of biomarker for diagnosis, prediction, and prognosis. To establish the efficacy of *PVT1*, validation in a large prospective study is required. The key to personalized medicine in colorectal cancer relies on an integrated understanding of genomic, transcriptomic, and proteomic data, to establish a biomarker panel with sufficient sensitivity and specificity to guide clinical decision making.

## Figures and Tables

**Table 1:** Summary of evidence for the role of PVT1 and miRNAs in promoting colorectal cancer.

miRNA	Role of PVT1	Proposed Pathogenesis Pathway	Reference
miRNA-146a	Decreases levels of miRNA-146a. rs13281615 G > A polymorphism on PVT1 and rs2910164 C > G polymorphism on miR-146a leads to favourable prognosis in CRC	PVT1/miRNA146a/COX2	[150]
miRNA-128	PVT1-214 upregulates Lin28 by competing for miRNA 128. let-7 is downregulated	PVT1-214/Lin28/let-7 axis	[137]
miRNA-216a-5p	PVT1 downregulates miRNA-216a-5p and reverses tumour suppressive effect in CRC	PVT1/miRNA-216a-5p/YBX1 axis	[151]
miRNA-455	PVT1 negatively regulates miRNA-455 and upregulates RUNX	RUNX2/PVT1/miRNA-455 regulatory axis	[152]
miRNA-214-3p	PVT1 downregulates miRNA-214-3p promoting CRC progression	PVT1/miRNA-214-3p/Insulin Receptor Substrate 1/PI3K/Akt	[153]
miRNA-455-5p	rs1252200336 polymorphism in PVT1 with ID/DD genotype leads to worse survival in CRC affecting Han Chinese population	PVT1 suppresses miRNA-455-5p and miR-455-3p	[143]
miRNA-30d-5p	PVT1 suppresses miRNA-30d-5p whilst upregulating RUNX2	PVT1/miRNA-30d-5p/RUNX2 axis	[154]
miRNA-26b	PVT1 inhibits miRNA-26b in promoting proliferation and metastases in CRC	PVT1/miRNA-26b	[132]
miRNA-145	PVT1 downregulation via sponging of miRNA-145 promotes CRC metastases	PVT1/miRNA-145 pathway	[155]
miRNA-16-5p	PVT1 binds to miR-16-5p to promote cell proliferation, migration and invasion through VEGFA/VEGFR1/AKT pathway in CRC	PVT1-miR-16-5p/VEGFA/VEGFR1/AKT axis	[156]
